# Supplementary material for: Using experimental gaming simulations to elicit risk mitigation behavioral strategies for agricultural disease management
Source: PLoS One. 2020 Mar 17;15(3):e0228983. doi: 10.1371/journal.pone.0228983 (PMC7077803; doi:10.1371/journal.pone.0228983)
Supplement: S1 Data — A demo of the game is hosted online at: (https://segs.w3.uvm.edu/demos/protocol). (ZIP) [file pone.0228983.s001.zip › Amendment .pdf]

The University of Vermont Committees on Human Research
Request for Modification/Amendment to Approved Protocol

Study modifications may not be instituted until you have received written approval from the Committee.

1. **CHRMS** [ ] **CHRBSS** [x] **#:** **16-232** **Principal Investigator (PI):** **Scott Merrill**

Protocol/Project Title: A human behavioral approach to reducing the impact of livestock pest or disease incursions of socio-economic importance (part 1) (CHRBSS: 16-232)

**Contact Name:**	**Scott Merrill**	**Contact Email:**	**Scmerril@uvm.edu**

2. **Date of Protocol Amendment:** **4/13/17** **Sponsor's Study #** [ ] **Sponsor's Amendment #** [ ]

Date of Consent Version (if applicable) [ ]

3. **Request for Revisions**

**A. Did IRB staff specifically request that you submit this amendment?**	x	**Yes**		**No**
**B. Is this protocol a Cancer Center protocol (involve the study of cancer)?**		**Yes**	x	**No**
**i. If yes, provide the date the amendment was approved by the Protocol Review and Monitoring Committee of the Cancer Center.**				
**C. Is this protocol a Clinical Research Center protocol?**		**Yes**	x	**No**

If yes, make sure that you have submitted this amendment to that Committee as well.

D. Check all that are applicable, explain in E. below, and attach supporting documentation.

x	Scientific Changes to Protocol		Change in Study Title
	Eligibility/Ineligibility Criteria Changes	x	Changes to Consent/Assent Form (submit revised consent form and consent addendum or information sheet if applicable)
x	Change in Protocol Procedures	x	Changes to Information Sheet or other previously approved materials given to subjects for information purposes
	Change Requiring Re-consent		Closure to Accrual (e.g. interventions and/ or follow-up still occurring locally)
	Study Suspension		Change in Sponsor (contact the office if you have a new or additional sponsor)
x	Addition of surveys/questionnaires, etc.		Change in Collaborating Sites (may need agreement, see manual guidance.)
	Change to surveys/questionnaires, etc.		Request for Review of Recruitment Materials
x	Change in Compensation		Request to Share Data/Specimens with another intuition (a data use or material use transfer agreement maybe required)
	Increase in Accrual Target to:	x	Other – Describe below in E.

E. Provide a description and justification for the requested change(s) listed in Section D. above.

Scientific Changes to Protocol / Change in protocol procedure:
We intend to start running experimental games in an online environment. This will allow us to increase our sample size significantly. The online environment does present different challenges. We have digitized our information sheet. Participants will read through the information sheet and click a button to "consent". Alternatively, they can click a button to re-read the information sheet. Because this is an online environment, they may choose to walk away from their computer at any time if they choose. Sample size is expected to increase up to 10,000 participants.

Addition of surveys/questionnaires:
After game play, participants will answer a series of questions. Questionnaire is attached.

Change in compensation:
As before compensation is dependent upon game play. However, we anticipate that games may be shorter and in an online environment. Many of our subjects will be recruited using Amazon Mechanical Turks. Participants using Amazon Mechanical Turks will be compensated at a rate of approximately $10 / hour.

Changes to consent/assent form / Changes to Information Sheet of other previously approved materials given to subjects for information purposes:
There are some minor revisions to the information provided by the information sheet. See attached for revised information sheet.

Additionally, because the research study will now be conducted online, delivery of the information sheet will change. The information sheet will be conveyed to the participant via a series of computer screens followed by a button labeled "I consent to participate in this research study" or users can select the "I would like to review the information sheet button" to repeat the information sheet presentation.

Other
Two other universities are currently in process to collect data using our experimental research study games. These studies will follow currently approved protocols. Each of these two university's research groups is going through the IRB approval protocols at their individual universities. Because we would like to share data, we would like to include them on our IRB. The PIs and contact details are as follows:

a. Timothy L. Sellnow, Ph.D.
   Office #226
   Nicholson School of Communication
   University of Central Florida
   Orlando, FL 32816-1344
   (407)823-3143.
   See attached for details.
b. Jason M. Martin, Ph.D.
   Assistant Professor
   Department of Communication Studies
   University of Missouri-Kansas City
   816-235-6347
   IRB approval pending

F. Grant Funding Changes

The IRB has the following regulatory mandate:
"Department of Health and Human Services (HHS) regulations at 45 CFR 46.103(f) require that each application or proposal for HHS-supported human subject research be reviewed and approved by the Institutional Review Board."

To meet this mandate, the IRB has to be made aware of when originally pending (including JIT) grants have subsequently been awarded or when supplements have been awarded. This gives us the opportunity to review the subsequent version of the grant in respect to the currently approved protocol. It is at this point that you need to revise your consent, if applicable, to include the new sponsor.

Check the Appropriate Funding Change Below

☐ Resubmission of Grant that was not previously funded *(Note: If this is a new competing grant or a renewal, an amendment is not appropriate, a new protocol is required.)*

☐ Original Grant has now been funded

☐ Supplemental Grant funding has been received

If any of the options above are checked, provide the specific grant information below.

InfoEd Proposal #	
Grant # *(full number including the version # related to the submission)*	

Is this new grant identical to the originally approved grant/protocol? ☐ Yes ☐ No

If yes, skip to #10 below. If no, explain the changes below and formally request an amendment to the protocol by completing this form. *(Note: Now is when you would need to revise your consent, if applicable, to include the new sponsor.)*

Confirm that the new corresponding grant is attached to this submission. ☐ Confirmed

☐ **Grant Funding has ended**

If so, and you are still using a consent form, you must remove the reference to the old sponsor and put in the new one. List the new sponsor below and the InfoEd number, if applicable.

G. Supporting sponsor documentation for this amendment is attached. ☐ Confirm by checking here.

4. **Is this change being submitted as a result of safety information already submitted to the IRB?**
☐ **Yes** ☒ **No**

If yes, provide the date of the safety information and a brief description of the safety information: ***(example: IDB submitted on 1/1/2001 with increased risk of seizures)***

5. **Does the proposed change affect the risk to subjects, either increase or decrease?**
☐ **Yes** ☒ **No**

If yes, please explain:

NOTE: Any change in research procedures that has active federal sponsorship that could result in an increased risk to human subjects will require prior NIH approval before implementation. Find guidance here http://grants.nih.gov/grants/guide/notice-files/NOT-OD-12-129.html

6. **After review of the proposed change, in the opinion of the Investigator, does the currently approved consent form require revision in order to adequately convey the potential risks of study participation?** *If yes, remember to attach a highlighted and a clean copy of a fully revised consent form for new subjects.*
☐ **Yes** ☒ **No**

If yes, please explain:

7. **Are there subjects currently enrolled?** ☐ **Yes** ☒ **No**

If yes, describe the process for re-consent and indicate how many participants you anticipate re-consenting. You need to develop and attach a consent addendum for review.

What is your approximate timeframe for informing current subjects? *Failure to inform subjects in a timely manner may be considered noncompliance depending upon the new information.*

8. Additional Comments:

9. Principal Investigator Signature ______________________ **Date** __________
