# Supplementary material for: Using experimental gaming simulations to elicit risk mitigation behavioral strategies for agricultural disease management
Source: PLoS One. 2020 Mar 17;15(3):e0228983. doi: 10.1371/journal.pone.0228983 (PMC7077803; doi:10.1371/journal.pone.0228983)
Supplement: S1 Data — A demo of the game is hosted online at: (https://segs.w3.uvm.edu/demos/protocol). (ZIP) [file pone.0228983.s001.zip › Exemption Certification 4_13_17.pdf]

The University of Vermont
Committees on Human Subjects
Serving the University of Vermont
and the UVM Medical Center

RESEARCH PROTECTIONS OFFICE
213 Waterman Building
85 South Prospect Street
Burlington, Vermont 05405
(802)656-5040 ph
www.uvm.edu/irb/

Protocol Exemption Certification

TO:	Scott Merrill, Ph. D.
FROM:	Gale Weld, Research Review Analyst, CIP
DATE OF REVIEW:	14-Apr-2017
DATE OF AMENDMENT:	13-Apr-2017
SUBJECT:	CHRBSS: 16-232
	A Human Behavioral Approach to Reducing the Impact of Livestock Pest or Disease Incursions of Socio-Economic Importance (Part 1)

Thank you for submitting the proposed modification to this exempt project. Following IRB review, it has been determined that the project still qualifies for exemption as indicated below.

Exemption Category: 2
Federal Exemption: "Research involving the use of educational tests (cognitive, diagnostic, aptitude, achievement), survey procedures, interview procedures, or observation of public behavior, unless: (a) information obtained is recorded in such a manner that human subjects can be identified, directly or through identifiers linked to the subjects; and (b) any disclosure of the human subjects' responses outside the research could reasonably place the subjects at risk of criminal or civil liability or be damaging to the subjects' financial standing, employability, or reputation."

This exemption is effective for the duration of the project UNLESS modifications are made that affect the original determination of exemption.
