# Supplementary material for: Using experimental gaming simulations to elicit risk mitigation behavioral strategies for agricultural disease management
Source: PLoS One. 2020 Mar 17;15(3):e0228983. doi: 10.1371/journal.pone.0228983 (PMC7077803; doi:10.1371/journal.pone.0228983)
Supplement: S1 Data — A demo of the game is hosted online at: (https://segs.w3.uvm.edu/demos/protocol). (ZIP) [file pone.0228983.s001.zip › Protocol Exemption Review and Determination.pdf]

The University of Vermont Committees on Human Research
Protocol Exemption Review and Determination

DATE STAMP		PROTOCOL NUMBER
		16-232

1. Protocol Title

A human behavioral approach to reducing the impact of livestock pest or disease incursions of socio-economic importance (CHRBSS: 15-319)

Principal Investigator (PI): Scott C Merrill **Degree:** Ph.D.

Checklist to Determine Whether Submission Meets Exemption Criteria

A. Does the project involve intervention or interaction with prisoners? [ ] Yes [x] No

If yes, stop. You must apply for either an expedited or full committee review.

B. Do you plan to use deception in the project? [ ] Yes [x] No

If yes, this project <u>may</u> require expedited or full committee review. Contact the office for guidance.

C. Does the project involve intervention or interaction with any of the following vulnerable populations? [ ] Yes [x] No

Cognitively Impaired	Mentally Ill	Pregnant Women
Wards of the State	Non-English Speaking	Fetuses

If yes, stop as regulations require that IRBs give special consideration for particularly vulnerable participants. Contact the office for guidance.

D. Will your research involve children under age 18? [ ] Yes [x] No, continue to E.

If yes, indicate below all the criteria under which exempt research is allowable with children.

- [ ] the research involves normal educational practices, or
- [ ] the project involves use of educational tests, or observation of public behavior when the investigator(s) do not participate in the activities being observed, or
- [ ] collection or study of existing data or specimens, when the data are recorded in such as manner that subjects cannot be identified, or
- [ ] collection or study of existing data or specimens, when the data are identifiable and the project is not federally funded.(Non-Federal Exemption #7)

Please explain why you feel that the inclusion of minors meets the criteria you have chosen.

If none of these situations apply, stop. You must apply for either an expedited or full committee review.

E. Will the project utilize educational tests, survey procedures, interview procedures, observations of public behavior?

[x] Yes, continue. [ ] No, continue with F.

Is the information obtained recorded in such a manner that human subjects can be identified, directly or through identifiers linked to the subjects; **<u>AND</u>**

Would any disclosure of the human subjects' responses outside the research reasonably place the subjects at risk of criminal or civil liability, or be damaging to the subjects' financial standing, employability, or reputation, or deals with sensitive aspects of the subject's own behavior, such as illegal conduct, drug abuse, sexual behavior or the use of alcohol.

[ ] Yes [x] No, continue with F.

If yes, stop. You must apply for either an expedited or full committee review.

F. Will the project use existing or archived data, documents, records, or specimens (in existence at the time of this protocol submission) that are collected in such a manner where subjects <u>cannot</u> be identified?

[ ] Yes, continue to 2. [x] No, continue to G.

G. Will the project use existing or archived data, documents, records, or specimens (in existence at the time of this protocol submission) that are collected in such a manner that subjects <u>can</u> be identified?

[ ] Yes [x] No

If yes, is this project federally funded? [ ] Yes [ ] No, continue

If yes, stop. You must apply for either an expedited or full committee review.

2. Principal Investigator Information

Dept.	Plant and Soil Sciences	Contact Phone:	802-656-0711	E-Mail:	Scmerril@uvm.edu

Campus/Office Address:	217 Jeffords Bldg, 63 Carrigan Dr
PI's Dept. Chair(s)	Deborah Neher
Date of Human Subjects Tutorial Completion	August 29, 2014

Is PI UVM Employee? Yes [x] No [ ] Is PI UVM Medical Center Employee? Yes [ ] No [x]

Is PI a Fellow, Resident, or Student? Yes [ ] No [x]

Please check graduate status if applicable: [ ] Graduate [ ] Undergraduate

Faculty Advisor/Sponsor (if fellow, resident, or student research)

Name:		Title:		Phone:	
Dept/Address:				E-mail:	
Date of Human Subjects Tutorial Completion					

NOTE: *Under normal circumstances only UVM or UVM Medical Center individuals can be PI. If you are not affiliated with either UVM nor UVM Medical Center, you must stop here and contact the RPO office for additional guidance.*

Do you want to appoint a primary contact other than the PI? Yes [ ] No [x]

Investigators wishing to appoint a contact for **all** IRB communications should complete the contact information requested below. **Primary contacts are considered "key personnel" and must complete required human subjects training.**

Contact Full Name		Email	
Department /Address		Phone	

3. Other Key Personnel

Definition: All individuals who will have contact with subjects or with research data locally.

➔ **It is strongly suggested that key personnel take our human subjects in research training.**

1	**Caitlin Danehy**	**3**	**Ahmed Hamed**
2	**Serge Wiltshire**	4	

4. Brief Lay Language Summary *(Use non-technical language that would be understood by nonscientific IRB members to summarize the proposed research project. The information must include: (1) objectives or aims, and (2) a brief but specific description of the procedure(s) involving the human subjects. Do not exceed one single-spaced 8 ½ X 11" page.*

This study will be one of many that will be a part of CHRBSS 15-319.

The purpose of the study is to better understand how people make decisions regarding biosecurity in our animal production industry (e.g., Dairy, Beef and Hog production) when various types of information and uncertainty are provided to them. For example, are producers more likely to adopt good management practices for the prevention of diseases in our animal industries if they are aware that there is a significant threat to their herds as contrasted to having no information about a potential disease. We intend to test the Theories of Planned Behavior and Utility Maximization. Of specific interest is the influence of Perceived Behavior Control, which suggests that individuals are more likely to engage in a behavior if they believe that they can influence the results. For example, one is more likely to learn how to ski if they believe that they have the athletic ability to learn how to ski (i.e., if one's perception indicates that they have control over the result, they are more likely to engage in the behavior). We intend to gather data to test these theories using experimental game research studies. We intend to recruit participants to play experimental games with an approximate duration of 1-2 hours. Games will run on computers. Computer games will simulate participation in the animal industries with player's choices impacting livestock health and economic return. For example, a participant may have three options during one round with options being 1) Increase mandatory sanitation practices, 2) Vaccinate your animals, or 3) Do nothing this round. Participants would then make a decision. All decisions or choices made during game participation would be considered the data collected to help answer our questions. Participants will be asked to respond to a series of scenarios that you will be framed around different environmental conditions and different levels of uncertainty. Participants will be compensated based on their responses and the resulting economic ramifications of those decisions. The risk for participating in this study is minimal. The information being collected will be coded to protect participant's identity and protect against an accidental breach of confidentiality. Participants have no greater risk from the study than one would from doing a similar amount of routine paperwork in a similar setting. Participants may withdraw from the study at any time.

5. Does the research involve the study of cancer or is it cancer-related? Yes [ ] No [x]

If yes, this research is also subject to a separate review by the University of Vermont Cancer Center. Click here, Protocol Review Committee(PRC) ,for the requirements.

6. Source of Support

a. Do you have any source of support for this project?

[ ] No, this is a research requirement with no monetary or other support.
[x] Yes, check below all that apply.

[ ] **Internal (Dept, Honors College, Pilot funds)** Specify [ ]
[x] **Project processed through Sponsored Project Administration (SPA)**

(e.g. NIH, DOD, cooperative groups, other state or local ,private foundations, etc.)

Name of Funding Agency	**USDA CAP**
InfoEd Proposal #	29034

7. Participant Information

a. How many participants are anticipated to enroll locally? [300]

Note: This requested number should equal the number of individuals, medical records, or specimens necessary.

b. Identify the targeted classes of subjects (check all that apply)

[x] Male or [x] Female
[x] Adults, provide age range [18-65] or [ ] Minors, provide age range [ ]
[x] Healthy or [ ] Persons with a specific disorder list disorder [ ]

c. Will participants be compensated? Yes [x] No [ ]

If yes, explain which participants in your pool will be compensated and the payment method. (e.g. cash, gift cards)

> Participation compensation with a minimum of approximately $15 and a maximum of approximately $30 depending upon experimental game decisions. Payments will be made in cash at time of experiment.

Provide monetary value of compensation and specify funding source. *Note: Principal investigators are not allowed to use their own personal funds to compensate participants.*

> Payments will range from $15-30 depending upon participant's game decisions. Funds are allocated in USDA CAP InfoEd Proposal# 29034

8. Recruitment

a. Summarize process of recruiting potential subjects. Specify inclusion and exclusion criteria when relevant. *If you are recruiting anyone who is subordinate to you (students or employees), additional protections may be necessary. Please contact the office.*

> We will be recruiting from US adult citizens. See attached recruitment materials.

*Attach any recruitment announcements such as flyers, or other advertisements.

b. Provide the specific name of the schools, country, clinic, or other agency from which subjects will be recruited and/or where research procedures will be conducted.

> Recruitment is not specific to an institution

*Provide letter(s) of support or permission letter(s) from the other agency(ies).

9. Exemption Categories and Procedures

a. Check the Federal Exemption Category or the Non Federal Exemption Category that you are applying for and complete the related questions.

NOTE: Cancer-related studies, although exempt from IRB review, MAY not be exempt from Vermont Cancer Center Protocol Review Committee review. Please refer to their website for further information on submission criteria.

[2] **Exemption 1, 2, & 3 - Interviews, Surveys, Audiotaping, Observation**

Exemption #1: Normal Educational Practices and Settings

Research conducted in established, or commonly accepted educational settings, involving normal educational practices, such as

(i) research on regular and special education instructional strategies, or

research on the effectiveness of, or the comparison among, instructional techniques, curricula, or classroom management methods.

Exemption #2: Educational Tests, Surveys, Interviews, or Observations

Research involving the use of educational tests (cognitive, diagnostic, aptitude, achievement), survey procedures, interview procedures, or observations of public behavior, unless:

(i) information obtained is recorded in such a manner that human subjects can be identified, directly or through identifiers linked to the subjects; and
(ii) any disclosure of the human subjects' responses outside the research could reasonably place the subjects at risk of criminal or civil liability, or be damaging to the subjects' financial standing, employability, or reputation, or deals with sensitive aspects of the subject's own behavior, such as illegal conduct, drug abuse, sexual behavior or the use of alcohol.

***Note: This exemption does not apply to research involving minors except for research involving educational tests or observation of public behavior when the investigator(s) do not participate in the activities being observed. All other research projects with minors require either expedited or full committee review.**

Exemption #3: Identifiable Subjects in Special Circumstances
Research involving the use of educational tests (cognitive, diagnostic, aptitude, achievement), survey procedures, interview procedures, or observations of public behavior that is not exempt under exemption #2, if:
(i) the human subjects are elected or appointed public officials or candidates for public office; or
Federal statute(s) require(s) without exception that the confidentiality of the personally identifiable information will be maintained throughout the research and thereafter.

If exemptions 1, 2 or 3 complete questions below.

i. Describe all procedures which will be completed during this study	**Participants will play an experimental game. Decisions by participants will be recorded as data to analyze for human behavior signals. Participants will make decisions on a computer by touching a screen, entering a number, or selecting different options.**
ii. Time estimation for subjects to complete procedures	**1-2 hours**
iii. Frequency of procedures	**The number of decisions per experimental game are expected to vary and range from approximately 50 to 200 rounds**
iv. Describe potential risks (e.g. accidental breach of confidentiality) and efforts to reduce any risks or discomforts	The risk for participating in this study is minimal. The information being collected will be coded to protect your identity and the potential risk for an accidental breach of confidentiality. You have no greater risk from the study than you would from doing a similar amount of routine paperwork in a similar setting.
v. Will photos, audio recordings, images be made during the study? Explain	**No**
vi. Describe procedures to secure all research data. How long will it be maintained?	**Data will be secured in a double password protected server. Personal identifiers are not needed for this research and thus will not be retained. Data will be made public after publication and will be stored in a data repository after completion of the project.**
vii. Describe plan for follow-up if necessary	**Participants will be provided with an information sheet providing contact information. Results will be disseminated after publication upon request. See attached information sheet.**

*Attach a copy of questionnaires, rating scales, or other instruments to be used.

☐ **Exemption #4: Use of Existing Data (unidentifiable)**
Research involving the collection or study of existing data, documents, records, pathological specimens, or diagnostic specimens, if these sources are publicly available or if the information is recorded by the investigator in such a manner that the subjects cannot be identified, directly or through identifiers linked to the subjects. **Note: This may not constitute "human subjects research" if the investigators/collaborators will not have access to the identities of the subjects. See guidance on Research Involving Coded Private Information or Biological Specimens.**

If using existing data, is the data publicly available?
☐ No ☐ Yes If yes, skip to question 10.
If the data or specimens will have any associated personally identifying information, skip to Non-Federal Exemption #7 below. If not, continue.

i. Identify the time period that the data/specimens were collected (be specific e.g. 1/1/2013 – 12/31/14)	
ii. Describe in detail the existing data/specimens to be collected for this research aim	
iii. Describe the source of the data/specimens	
iv. Under what authority do you now have access to the data/specimens (explain if there was prior consent to the use of the data/specimen for research)	
v. Describe potential risk and efforts to reduce risk	

vi. Describe how the research data will be recorded by the investigator(s) in such a manner that the subjects cannot be identified, directly or through identifiers linked to the subjects. (not coded in any way)

☐ **Exemption #5: Public Benefit or Service Programs**

Research and demonstration projects which are conducted by or subject to the approval of the [Federal] Department or Agency heads, and which are designed to study, evaluate, or otherwise examine:

(i) Public benefit or service programs;
(ii) procedures for obtaining benefits or services under those programs;
(ii) possible changes in or alternatives to those programs or procedures; or
(iii) possible changes in methods or levels of payment for benefits or services under those programs.

☐ **Exemption #6: Taste and Food Evaluation and Acceptance Studies**

Taste and food quality evaluation and consumer acceptance studies,

(i) if wholesome foods without additives are consumed or
(ii) if a food is consumed that contains a food ingredient at or below the level and for a use found to be safe, or agricultural chemical or environmental contaminant at or below the level found to be safe, by the Food and Drug Administration or approved by the Environmental Protection Agency or the Food and Safety and Inspection Service of the U.S. Department of Agriculture.

☐ **Non-Federal Exemption #7 - Research involving collection or study of existing data, documents, records, pathological specimens or diagnostic specimens, when this information is personally identifiable or coded.**

i. Do you have federal funding for this project? ☐ Yes ☐ No

If yes, **stop**. You must apply for either an expedited or full committee review, otherwise continue

i. Identify the time period that the data/specimens were collected (be specific e.g. 1/1/2013 – 12/31/14)	
ii. Describe in detail the existing data/specimens to be collected for this research aim	
iii. Describe the source of the data/specimens	
iv. Under what authority do you now have access to the data/specimens (explain if there was prior consent to the use of the data/specimen for research)	

v. Describe potential risk and efforts to reduce risk

vii. Describe how the research data will be recorded by the investigator(s) in such a manner that protects and ensures subjects' confidentiality (i.e. master list with link to a code separate from the coded data).

10. Consent and HIPAA

a. Are you requesting a Waiver of Informed Consent? [ ] Yes [x] No

This request means that you will not be obtaining verbal, nor implied, nor written consent.

If yes, does the project meet the following criteria necessary to allow for a waiver of consent?

-Involves no more than minimal risk
-Will not adversely affect the rights and welfare of subjects.
-Research cannot be conducted without the waiver of consent
AND
-Whenever appropriate the subjects will be provided information once the research is complete.

Yes [ ] If yes, continue.
No [ ] If no, a consent process is necessary. Proceed to 9.c.

b. Are you requesting a Waiver of HIPAA Authorization? [ ] Yes [ ] NA

This only applies if you are collecting Protected Health Information (PHI) from a Covered Entity (hospital or other healthcare entity) thus making the data protected under the HIPAA regulations. This request means that you will not be obtaining verbal, nor implied, nor written authorization.

If yes, does the project meet all of the consent waiver criteria plus the following additional HIPAA criteria?

(a) The use or disclosure of PHI involves no more than minimal risk to the privacy of individuals, based on, at least, the presence of the following criteria:
(1) An adequate plan to protect the identifiers from improper use and disclosure;
(2) An adequate plan to destroy the identifiers at the earliest opportunity consistent with conduct of the research, unless there is a health or research justification for retaining the identifiers or such retention is otherwise required by law;
(3) Adequate written assurances that the PHI will not be reused or disclosed to any other person or entity, except as required by law, for authorized oversight of the research study, or for other research for which the use or disclosure of PHI would be permitted under the HIPAA privacy rule.
(b) The research could not practicably be conducted without the alteration or waiver; and;
(c) The research could not practicably be conducted without access to and use of the protected health information.

Yes [ ] If yes, continue to 11.
No [ ] If no, a consent process is necessary. Continue to 10.c.

c. Are you requesting a Waiver of Documentation of Informed Consent? [x] Yes [ ] No

This request means you are obtaining verbal or implied consent without obtaining the subject's signature on a consent form.

If yes, describe the consent process below:

An information sheet will be provided to each subject with time given to each subject to read the document. Verbal consent will then be requested from each participant (see attached information sheet for details).

*If an information sheet will be provided to the subjects, attach the sheet for review.

11. Location of Research Activities (complete all that apply)

UVM Medical Center

[ ] Main Hospital/ACC
[ ] Clinical Research Center
[ ] 1 South Prospect (UHC)
[ ] Other UVM Medical Center Location(s)
Specify location(s):

University Campus
Specify location(s):

School/School System
Specify location(s):

Correctional Facility
Specify location(s):

Other Location

Specify location(s): the Social Ecological Gaming and Simulation Lab (Jeffords 105a, UVM) and at external sites using our suite of mobile surface pro devices.

12. Assurances

Principal Investigator

As Principal Investigator of this study, I assure the Committees on Human Research that the following statements are true:

- I will seek prior approval of any changes to this exempt project which may change the original exemption.
- I will report any unanticipated problems.

[signature] 10/27/15

Original Signature of PI Date

Faculty Advisor/Sponsor (if applicable)

As the faculty advisor/sponsor for this protocol, I certify that

- I have reviewed the protocol and believe that it is scientifically and ethically sound;
- The student has the necessary training, experience and knowledge to conduct the research in a manner consistent with the regulations governing human subject research and sound research principles;
- I will oversee and monitor the conduct of this research by communicating regularly with the student investigator;
- Assist with any resolution of any problems or concerns encountered during the research;
- The UVM IRB is notified in the event of an adverse event or protocol deviation.

Is there is a thesis or dissertation committee reviewing this research? Yes ☐ No ☐

If yes, date of approval: ☐

Original Signature of Faculty Sponsor Date

Printed Name

Subject: RE: IRB proposed modification to CHRBSS_15-319
From: "Scott Merrill" <scott.c.merrill@uvm.edu>
Date: 10/27/2015 2:11 PM
To: "'Gale Weld'" <Gale.Weld@uvm.edu>

Hi Gale,

Attached is my Exempt cover form for a protocol under the CHRBSS 15-319 umbrella, signed signature page from the same form, information sheet and recruitment material.

Let me know how it looks.

Thanks for your help.

Cheers,

Scott Merrill

From: Gale Weld [mailto:Gale.Weld@uvm.edu]
Sent: Tuesday, October 13, 2015 11:06 AM
To: Scott Merrill <scott.c.merrill@uvm.edu>
Cc: 'Julie Smith DVM PhD' <julie.m.smith@uvm.edu>
Subject: Re: IRB proposed modification to CHRBSS_15-319

Scott:

In reviewing the material you submitted electronically.

The Protocol form is incomplete, but I believe this can be deemed as Exempt.

What I need for you to complete at this point is an Exemption Review and Determination request form.

See link to form noted below:

http://www.uvm.edu/irb/form/exemptcoverform.docx

This form will replace the "incomplete" protocol application pending review.

Thank you
gale

Gale A. Weld
Research Review Administrator

Research Protections Office
University of Vermont
Main Phone: (802)656-5040

http://www.uvm.edu/irb/

On 10/12/2015 2:02 PM, Scott Merrill wrote:

> Hi Gale,
>
> After a brief discussion with Julie Smith, it was agreed that I would send you a full Human Research Protocol form (Attached). This protocol is one of many that will fall under CHRBSS 15-319.
>
> Additionally, I have attached the information sheet, consent form and recruitment template that are specific to this protocol. Let me know what additional information that you would like to help complete this request.
>
> Cheers,
>
> Scott Merrill

Information Sheet

Title of Research Project: A human behavioral approach to reducing the impact of livestock pest or disease incursions of socio-economic importance

Social Ecological Project

Principal Investigator: Julie Smith

Sponsor: United States Department of Agriculture (USDA)

You are being invited to take part in this research study about how individuals make decisions. You must be 18 years of age or older to participate.

This study is being conducted by Scott Merrill, a Research Assistant Professor in the Plant and Soil Science Department at the University of Vermont

We encourage you to ask questions and take the opportunity to discuss the study with anybody you think can help you make the decision to participate in this research study.

Why is This Research Study Being Conducted?
The purpose of the study is to better understand how people make decisions regarding biosecurity in our animal production industry (e.g., Dairy, Beef and Hog production) when various types of information and information quality are provided to them.

How Many People Will Take Part In The Study?
Approximately 300 people will take part in this study.

What Is Involved In The Study?
You are being asked to take part in an experimental economics and behavioral theory study that will be run as a simulated game. You will be asked to respond to a series of scenarios that you will be framed around different environmental conditions and different levels of information uncertainty. Games will run on computers. Computer games will simulate participation in one of three livestock industries (pork, beef and dairy) with player's choices impacting livestock health and economic return.

Your participation in the game will last 1-2 hours in total.

What Are The Risks Of The Study?
The risk for participating in this study is minimal. The information being collected will be coded to protect your identity and the potential risk for an accidental breach of confidentiality. You have no greater risk from the study than you would from doing a similar amount of routine paperwork in a similar setting.

What Are The Benefits of Participating In The Study?

There are no substantial benefits to you from the research. By learning more about people's decision-making, we hope that the research will benefit society by increasing our understanding how individual behavior will impact economic status, health and management policy.

What Other Participation Options Are There?
The only other option is not to participate.

Are There Any Costs?
There is no cost to you other than your time.

What Is the Compensation?
You will be given a flat compensation of $15 for your participation. You will be afforded the opportunity to earn up to an additional $15 depending on your performance in the game.

Can You Withdraw From This Study?
You may discontinue your participation in this study at any time. Any intentional disruption of the study may result in being asked to leave.

What About Confidentiality?
All research information will be kept in a confidential form at the University of Vermont. The security of your information will be maintained by Scott Merrill. The results of this study may eventually be published, but your confidentiality will be maintained. All participants are asked to keep their responses to all parts of the experiment confidential.

Contact Information
You may contact Dr. Scott Merrill, Dr Chris Koliba or Dr Asim Zia, the Investigators in charge of this study, at 802-656-0711 or scmerril@uvm.edu for more information about this study. If you have any questions about your rights as a participant in a research project you should contact Nancy Stalnaker, the Director of the Research Protections Office, at the University of Vermont at 802-656-5040.

Statement of Consent
You have been given and have read or have had read to you a summary of this research study. Should you have any further questions about the research, you may contact the person conducting the study at the address and telephone number given below. Your participation is voluntary and you may refuse to participate or withdraw at any time without penalty or prejudice.

If you agree to participate in this study, then please state "yes, I agree to participate". This will be considered your verbal consent to take part in this research study.

Name of Principal Investigator for this research study: Scott Merrill

Address: University of Vermont;
217 Jeffords Hall. Burlington, VT 05405
Telephone Number: 802-656-0711

Social Ecological Gaming & Simulation Lab Research Study

Recruiting Now for January 23rd 1-3 pm session

How would you like to play games to win money? The Social-Ecological Gaming & Simulation (SEGS) lab at UVM is recruiting students to participate on January 23rd from 1-3 pm in a gaming research study by the supported by the United States Department of Agriculture and the University of Vermont. Participants will play games in a group setting for two hours and will have the chance to win up to $30 (minimum of $15) by playing! Participants will walk out of the door with cash in their hands!

If you like to play games, win money, and are interested in participating, please contact Scott Merrill at Scott.C.Merrill@uvm.edu or Courtney Hammond at crhammon@uvm.edu.

For questions or concerns, or if you would like to be put on a contact list for later gaming dates contact Scott Merrill at Scott.C.Merrill@uvm.edu.

Subject: Re: Exempt submission / Please comment regarding Compensation
From: Theodore Marcy <theodore.marcy@uvm.edu>
Date: 11/12/2015 3:50 PM
To: Gale Weld <Gale.Weld@uvm.edu>

> On Nov 12, 2015, at 2:58 PM, Gale Weld <Gale.Weld@uvm.edu> wrote:
>
> Hi Ted:
>
> Attached is an Exempt request. Please provide your comment regarding compensation.
>
> Explanation/Description:
>
> Today I reviewed the compensation process for this project.
>
> √ I agree that the process for compensation in this project is fair.
>
> □ I do not agree that the process for compensation in this project is fair.
>
> Comment: Contingent compensation based on decisions in an educational game exploring biosecurity decisions with compensation of between $15-$30 for a one to two hour participation time period
>
> Theodore W. Marcy, MD MPH

> Thank you.
>
> --
> Gale Weld
> Research Review Administrator
> Research Protections Office
> University of Vermont
> 213 Waterman Building
> Burlington, VT 05405
> Main Phone: (802) 656-5040
>
> http://www.uvm.edu/irb/
>
> <16-232 Exempt Submission (1).pdf>
