# Supplementary material for: Using experimental gaming simulations to elicit risk mitigation behavioral strategies for agricultural disease management
Source: PLoS One. 2020 Mar 17;15(3):e0228983. doi: 10.1371/journal.pone.0228983 (PMC7077803; doi:10.1371/journal.pone.0228983)
Supplement: S1 Fig — (PDF) [file pone.0228983.s002.pdf]

SEGS Lab

social ecological gaming and simulation

USDA

Welcome to the experiment. The following slide show will guide you through the simulation.

Press the enter/return key or touch the screen to advance.

This material is based upon work that is supported by the National Institute of Food and Agriculture, U.S. Department of Agriculture, under award number 2015-69004-23273.
Any opinions, findings, conclusions, or recommendations expressed in this publication are those of the author(s) and do not necessarily reflect the view of the U.S. Department of Agriculture.

Welcome to an experiment about the economics of decision making. In the course of the experiment, you will have several opportunities to earn money. Any money earned during this experiment will initially be recorded as experimental dollars. At the end of this experiment, we will convert your experimental dollars into actual US dollars. The more experimental dollars you earn the more actual US dollars you will receive at the end of the experiment.

At the end of the experiment, your earnings will be converted at a rate of $1 US dollar for $23,500 experimental dollars.

Overview

In today's experiment, you will participate in a number of rounds, each designed to mimic decisions made over the period of six months. Each round is independent, meaning that decisions during a round do not affect future rounds in any way. The only value that gets carried over across rounds is your cumulative profit, which will be used to calculate your payout at the end of the experiment.

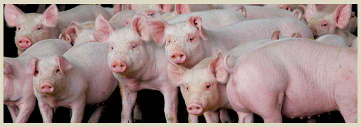

http://www.schickenterprises.com/visipork/

Pork Production

Today you will be operating as a pork producer, responsible for managing a hog production facility.

Infectious disease

An unnamed infectious disease, that is spread through the air, is a risk in the region of the country where your facility is located.

Distance from infection

Because the infectious disease is airborne, the farther your facility is from an infected facility the less likely your facility is to contract the disease.

Animal health protection protocols

Animal health protection protocols can be purchased to help reduce the likelihood of your animals catching a disease. Animal health protection protocols can only be increased one level per month; from None to Low, Low to Medium, then Medium to High. As more protocols are purchased, your facility's animal health **Protection Level** will increase. The higher the animal health **Protection Level** the less likely a facility's animals will become infected.

Improving your facility's animal health protection protocols costs money

- Part of your managerial role will be to decide how much to invest in animal health protection to attempt to keep your animals healthy.

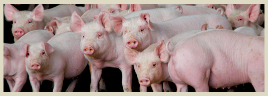

http://www.schickenterprises.com/visipork/

Infection Cost

- If your facility becomes infected, all your livestock will be culled, and you will lose all profits for that year.
- Upon infection, $25,000.00 USD will be subtracted from your score and the next round will begin

User Interface

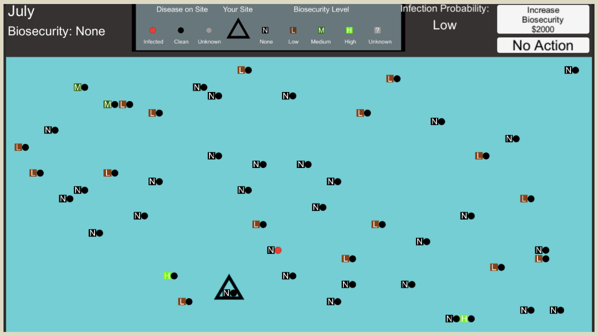

The upper box provides basic information, such as current month, current round profit, and your animal health Protection Level.

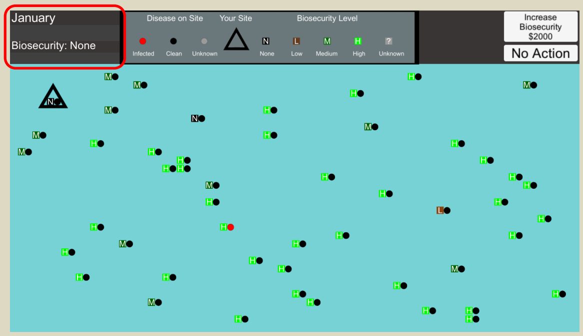

The black triangle surrounds your facility

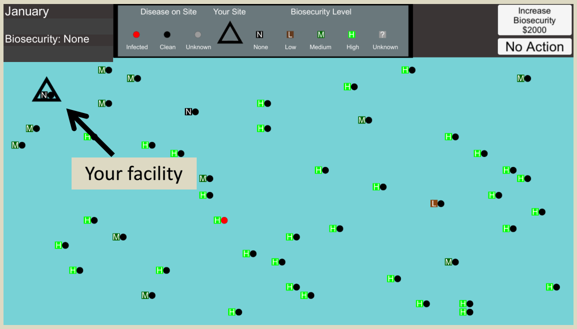

Also displayed is a map of the facilities in the region. The legend describes the two pieces of information that are available about each of the 50 facilities in your region.

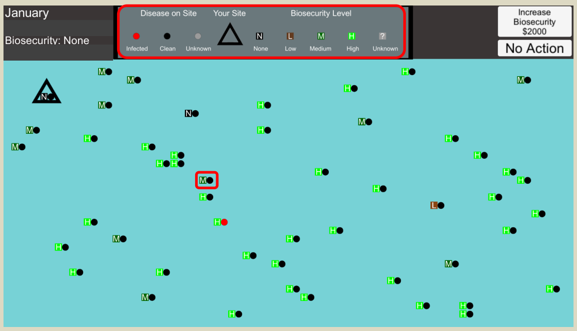

The two pieces of information are noted by a box and a circle. The box indicates animal health Protection Level and the circle indicates infection status.

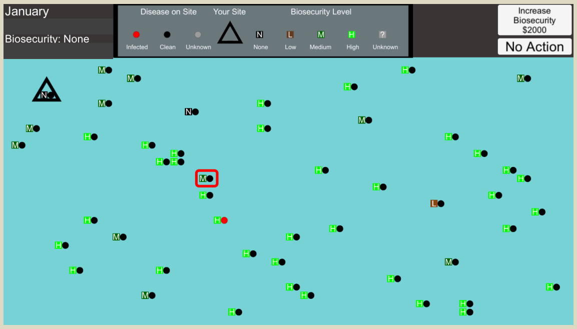

A black circle indicates that animals are not infected.

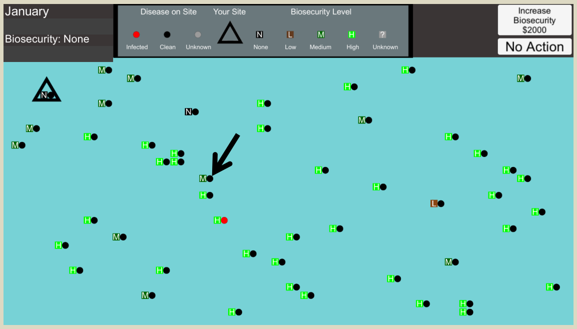

A red circle indicates that animals are infected.

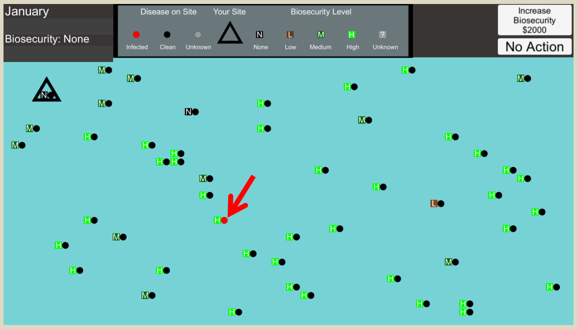

If your facility becomes infected, the current year will end and you will be prompted to start the next round.

Infection! Round Over
You earned $-20000 this Round
Total Profit: $78000

Press N for next Round

The color of the box changes from black (no protection) to brown to green as animal health Protection level increases (improves) by purchasing protection

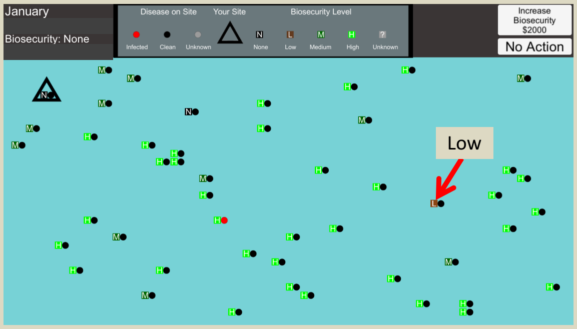

The color of the box changes from black (no protection) to brown to green as animal health Protection level increases (improves) by purchasing protection

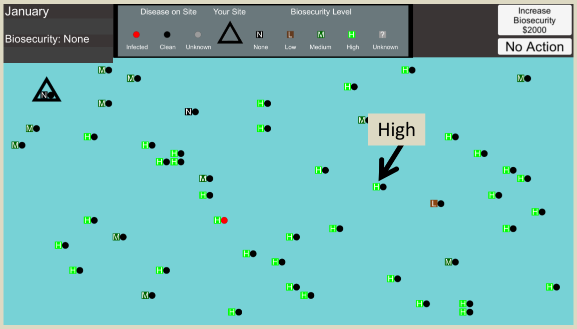

To increase your animal protection level, you can purchase protection using the button in the upper right corner. Recall that there is a maximum of one increase per month.

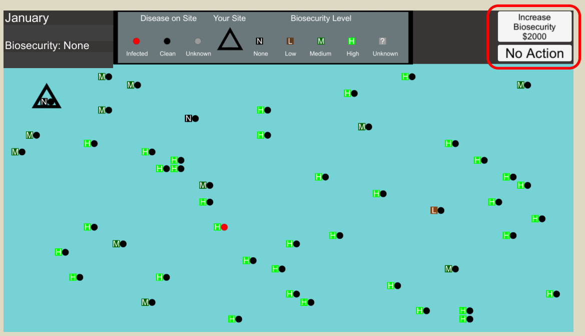

Practice rounds

- You will have one practice round (six-month period) to get used to playing the game
- The money you make or lose during the practice round will not be added to your experimental dollar totals

At the end of all rounds, you will be asked to complete a quick survey.

†
