# Supplementary figures and images for: Using experimental gaming simulations to elicit risk mitigation behavioral strategies for agricultural disease management
Source: PLoS One. 2020 Mar 17;15(3):e0228983. doi: 10.1371/journal.pone.0228983 (PMC7077803; doi:10.1371/journal.pone.0228983)

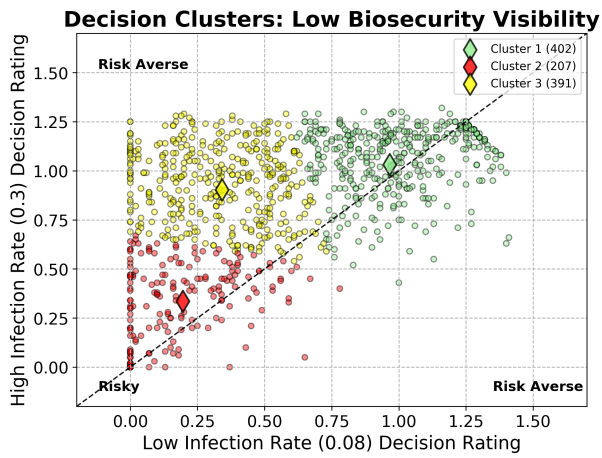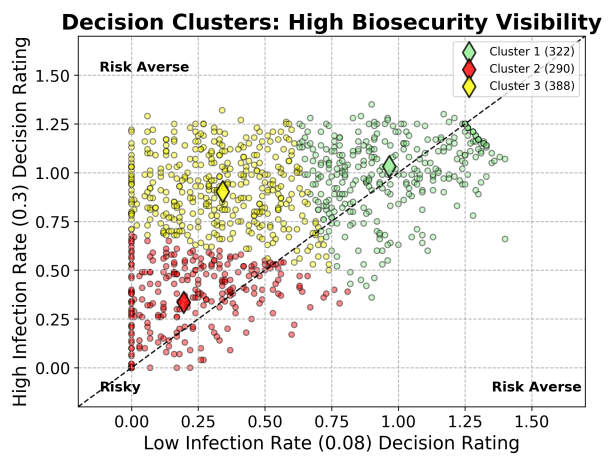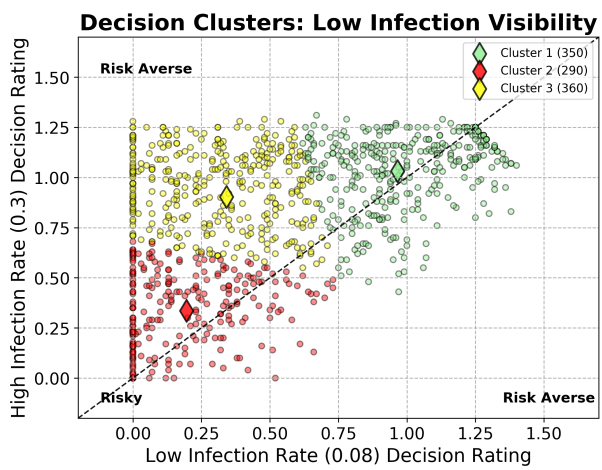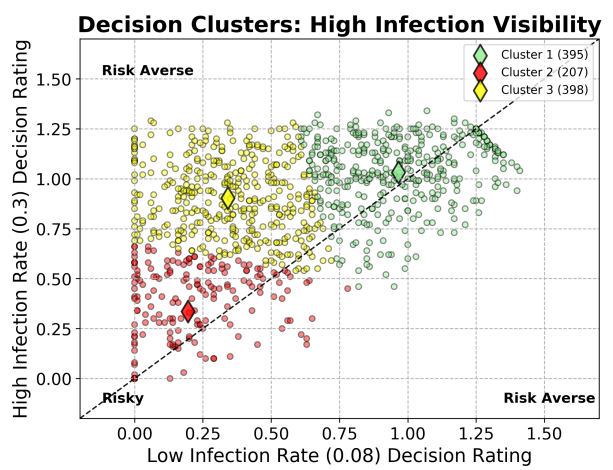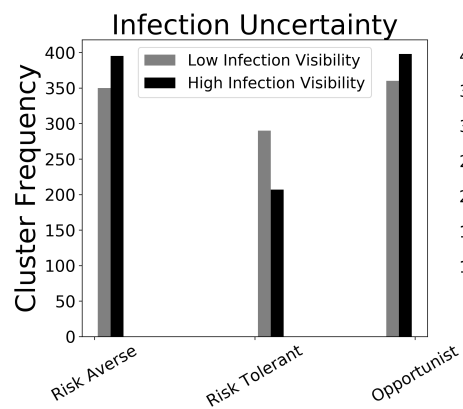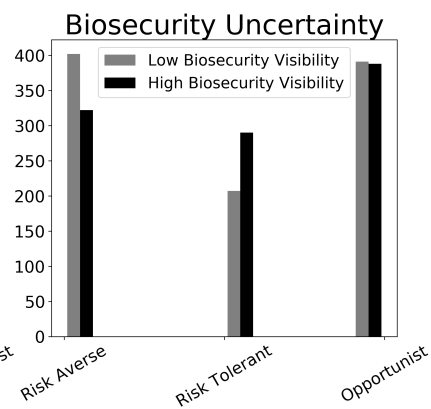

Supplement: S2 Fig — Participant biosecurity adoption ratings are clustered for each set of visibility treatments. Cluster 1 are Risk Averse (green), Cluster 2 are Risk Tolerant (red) and Cluster 3 are Opportunistic (yellow). The top row compares Low neighboring biosecurity visibility (i.e., high uncertainty) to high biosecurity visibility. The bottom row compares infection visibility treatments. We found a significant difference in the clustered risk distributions for biosecurity visibility treatments. Histograms explicitly show the cluster differences between information uncertainty treatments. (PDF) [file pone.0228983.s003.pdf]
